# Supplementation of branched-chain amino acids decreases fat accumulation in the liver through intestinal microbiota-mediated production of acetic acid

**DOI:** 10.1038/s41598-020-75542-3

**Published:** 2020-10-30

**Authors:** Masao Iwao, Koro Gotoh, Mie Arakawa, Mizuki Endo, Koichi Honda, Masataka Seike, Kazunari Murakami, Hirotaka Shibata

**Affiliations:** 1 Department of Endocrinology, Metabolism, Rheumatology and Nephrology, Faculty of Medicine, Oita University, 1-1 Idaigaoka, Yufu City, Oita Japan; 2 Department of Gastroenterology, Faculty of Medicine, Oita University, Oita, Japan

**Keywords:** Diseases, Gastroenterology

## Abstract

Non-alcoholic fatty liver disease (NAFLD) is a significant problem because its prevalence is increasing worldwide. Recent animal studies have identified gut microbiota as a potentially important player in the pathogenesis of NAFLD. Previously, we reported that the administration of branched-chain amino acids (BCAAs) reduces hepatic fat accumulation in experimental animal models. This study aimed to clarify how changes in the intestinal microbial flora following the administration of BCAAs affect a high-fat diet (HF)-induced fat accumulation in the liver. We examined whether the administration of BCAAs alters the development of hepatic fat accumulation as well as intestinal microbial flora. The oral administration of BCAAs (3% kcal) induced a significant increase in *Ruminococcus flavefaciens* (*R. flavefaciens*) and portal acetic acid levels, and it reduced hepatic fat accumulation in HF-fed rats. In addition, BCAAs reduced the expression of the lipogenesis-related genes *FAS* and *ACC* in the liver. Furthermore, we observed that *R. flavefaciens* is essential for promoting a BCAA-induced reduction in hepatic fat accumulation. These data suggest that BCAA treatment induces the proliferation of intestinal flora including *R. flavefaciens* and that portal acetic acid synthesized from intestinal flora improves NAFLD by downregulating the expression of FAS and ACC in the liver.

## Introduction

Non-alcoholic fatty liver disease (NAFLD) is a significant public health problem because of the dramatic increase in the prevalence of obesity. The accumulation of triglycerides in hepatocytes (hepatic steatosis) is the most common liver phenotype in NAFLD. Recently, the gut microbiota has gained great attention in metabolic diseases since gut dysbiosis has been demonstrated in patients with NAFLD as well as those with obesity and metabolic syndrome ^1,2^. A previous study identified gut microbiota as a potentially important player in the pathogenesis of NAFLD ^2^. A “multiple-hit hypothesis” has been proposed for the aetiology of NAFLD. In this model, various elements are posited to be involved in the development of the disease state, and the intestinal flora is identified as one of these elements ^3^. Moreover, changes in the amounts of lipopolysaccharides ^4,5^, bile acids ^6,7^ and short-chain fatty acids (SCFAs) ^8–11^ in association with intestinal bacteria have been suggested to influence the mechanism of NAFLD development.

The gut microbiota influences the host metabolic phenotype via a range of mechanisms, including the production of energetic substrates by fermentation, especially SCFAs such as acetic acid ^12^. Acetic acid is the main SCFA produced by several intestinal bacteria, and cellulose intake is essential for synthesizing acetic acid. In the field of gastroenterology, branched-chain amino acid (BCAA) preparations have mainly been used to improve hypoalbuminaemia and hepatic encephalopathy in patients with cirrhosis. In addition, through research using animal models and human subjects, BCAAs have been reported to inhibit carcinogenesis in hepatocytes and reduce oxidative stress ^13–16^. Previously, we reported that the administration of BCAAs in experimental animal models of NAFLD promotes fat consumption in muscle tissues and reduces hepatic fat accumulation ^17^. Little is known about the link between BCAAs and intestinal flora in humans, although the administration of BCAAs has been reported to increase specific bacterial species primarily within the stomachs of herbivores ^18–20^. Furthermore, no report has identified how changes in intestinal flora due to the administration of BCAAs for the treatment of NAFLD bring about its physiological changes.

The goal of the present study is to determine whether (1) there are specific compositional and functional characteristics of the gut microbiome in obese-induced fat accumulation in the liver, which may be associated with BCAA administration, using select microbial taxa found to be differentially abundant in next generation sequencing data, and (2) changes in intestinal flora as a result of BCAA administration is also related to portal acetic acid levels as well as fat accumulation in the liver.

## Results

### Summary of experimental plan “Design 1”

In the intestinal flora, BCAA supplementation increased the proportion of *R. flavefaciens* and portal acetic acid levels in animals subjected to a HF. Moreover, BCAA treatment induced the activity of AMPK which downregulated lipogenesis-related enzymes such as fatty-acid synthase (FAS) and acetyl-CoA carboxylase (ACC), and improved fat accumulation in the liver.

### Summary in experimental plan “Design 2”

In animals subjected to a high-fat diet, the BCAA-induced alterations in intestinal flora, portal acetic acid levels, AMPK activity, expression of lipogenesis-related enzymes, and hepatic fat accumulation was disappeared due to cellulose deficiency.

### Effect of BCAA supplementation on daily caloric intake, body weight, serum BCAA levels and biochemical data

In Design 1, there was no difference in body weight among all groups, although the daily caloric intake of the HF-BCAA group was significantly greater than that of a standard diet (ST)-fed group. In addition, there was no significant alteration in the serum BCAA, alanine aminotransferase (ALT), total cholesterol (TC), triglyceride (TG) and glucose levels among all groups (Supplementary table. S1). In Design 2, there was no difference in daily caloric intake, body weight, or serum parameters described above among all groups (Supplementary table. S2).

### Effect of BCAA supplementation on intestinal flora

**Figure. 1:**
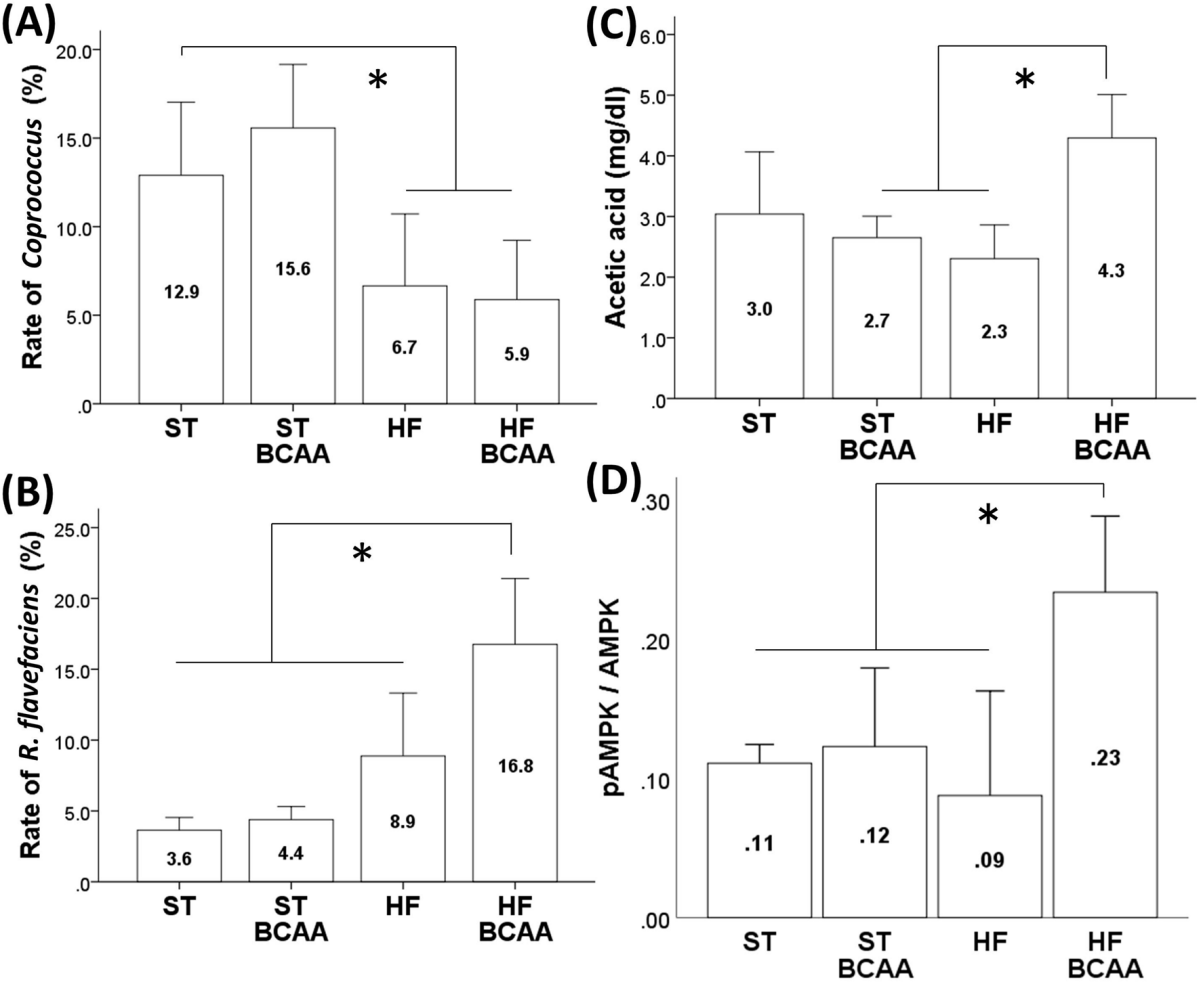
Effects of BCAA supplementation on the proportion of genus *Coprococcus* (**A**) and *R. flavefaciens* (**B**), portal acetic acid concentrations (**C**) and hepatic AMPK activity (**D**). The rate of intestinal flora was calculated from the number of sequenced leads. Values are expressed as the means ± S.D. *p < 0.05 between the groups connected by a line. ST, fed a standard diet; ST-BCAA, fed a ST with added BCAAs; HF, fed a high-fat diet; and HF-BCAA, fed a HF with added BCAAs.

We examined whether BCAA administration affects intestinal flora in Design 1 at the genus level and the species level (Supplementary figure. S1). At the genus level, there was no significant difference among the groups except that the proportion of the genus *Coprococcus* was significantly reduced following HF feeding regardless of BCAA supplementation (Fig. 1A, Supplementary fig. S1). At the species level in Design 1, there was no significant difference in the groups except that BCAA supplementation elevated the proportion of *Ruminococcus flavefaciens* (*R. flavefaciens*) in the HF feeding group but not in the ST feeding group (Fig. 1B, Supplementary fig. S1).

### Effect of BCAA supplementation on portal acetic acid levels

Moreover, BCAA administration increased portal acetic acid levels in the HF feeding group (HF group, 2.30 ± 0.28 mg/dl; HF-BCAA group, 4.29 ± 0.36 mg/dl) but not in the ST feeding group (ST group, 3.04 ± 0.51 mg/dl; ST-BCAA group, 2.65 ± 0.18 mg/dl; Fig. 1C).

### Effect of BCAA supplementation on the pAMPK/AMPK ratio in the liver

In the HF feeding group, a significant increase in the pAMPK/AMPK ratio was observed following BCAA treatment which elevated the portal acetic acid concentration, indicating that BCAA treatment activated hepatic AMPK in the HF feeding group (Fig. 1D).

### Effect of BCAA supplementation on hepatic fat accumulation and liver fibrosis

**Figure. 2:**
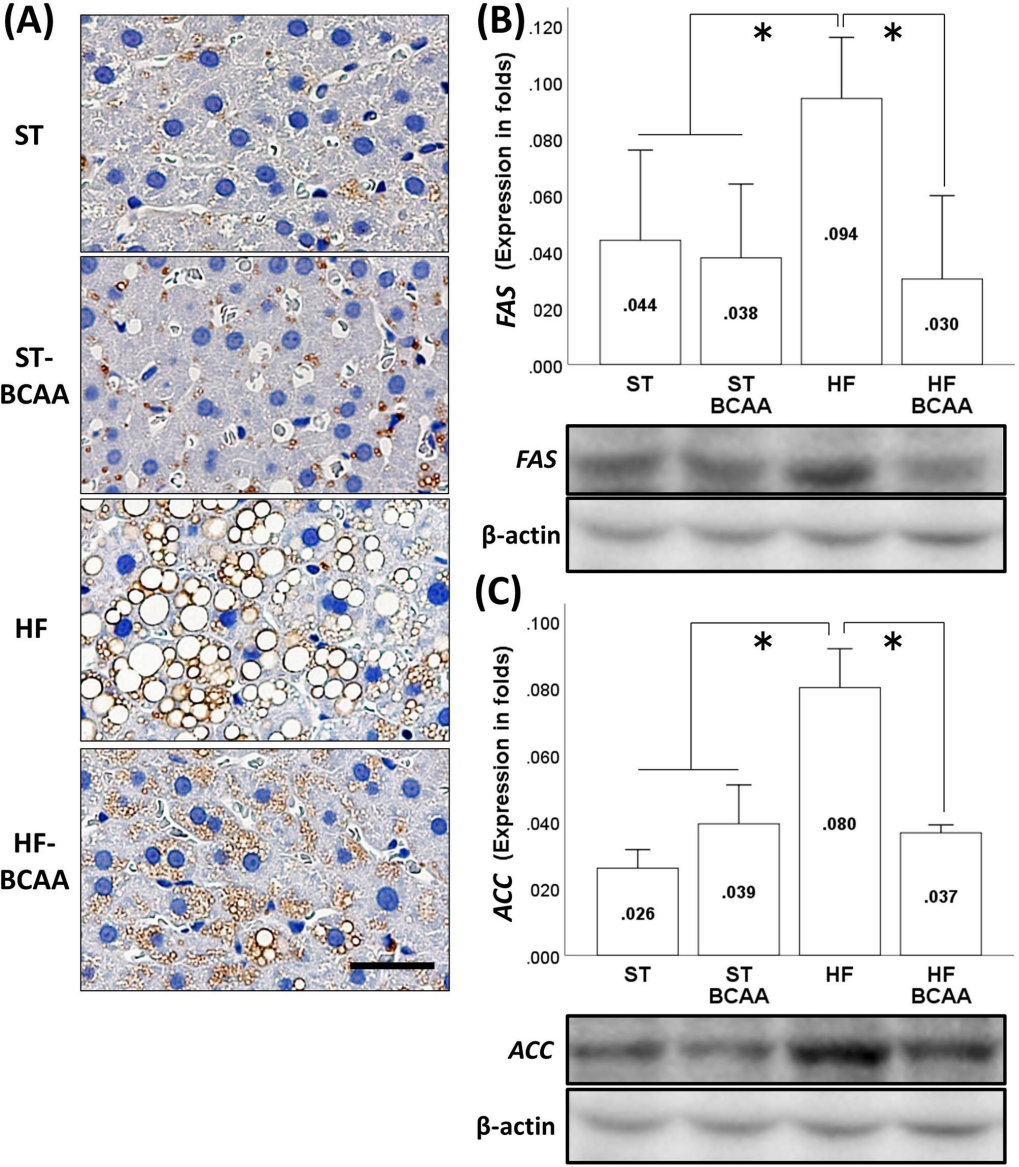
Effects of BCAA supplementation on HF-induced fat accumulation in the liver (**A**) and hepatic lipogenesis-related protein expression of FAS (**B**) and ACC (**C**). Representative adipophilin immunostaining of the liver (**A**). Values are expressed as the means ± S.D. (**B, C**). *p < 0.05 between the groups connected by a line. Scale bar = 20 μm. A representative band of each group was trimmed. Full-length gels are presented in Supplementary figure. S9. ST, fed a standard diet; ST-BCAA, fed a ST with added BCAAs; HF, fed a high-fat diet; and HF-BCAA, fed a HF with added BCAAs.

In addition, the morphological findings (Fig. 2A) and results of hepatic TG content analysis (Supplementary fig. S2) showed that BCAA treatment attenuated HF-induced fat accumulation in the liver, although these findings were not observed in the ST feeding group. We also calculated the NAFLD activity score for steatosis. BCAA administration decreased the HF-induced elevation in the steatosis score, indicating that BCAA treatment resulted in a significant improvement in liver steatosis in the HF feeding group (Supplementary fig. S2). However, there was hardly any liver fibrosis observed in each group (Supplementary fig. S2).

### Effect of BCAA supplementation on the expression of lipogenesis-related genes and proteins

We investigated whether BCAA administration altered the expression of lipogenesis-related genes, such as *FAS,* which encodes an enzyme that catalyses fatty acid synthesis, and *ACC,* which encodes an enzyme that catalyses fatty acid synthesis in the liver. Quantitative RT-PCR analyses showed that HF feeding increased the transcription of *FAS* and *ACC*, which were completely reversed by BCAA treatment (Supplementary fig. S3). We also evaluated the protein expression of FAS and ACC in the liver. Similar to the transcription data for these mRNAs, HF-induced an increase in the expression of FAS (Fig. 2B, Supplementary fig. S9) and ACC (Fig. 2C, Supplementary fig. S9) proteins, but they were was suppressed by BCAA supplementation in the HF feeding group.

### Effect of BCAA supplementation on intestinal flora and portal acetic acid levels in cellulose-deficient HF feeding conditions

**Figure. 3:**
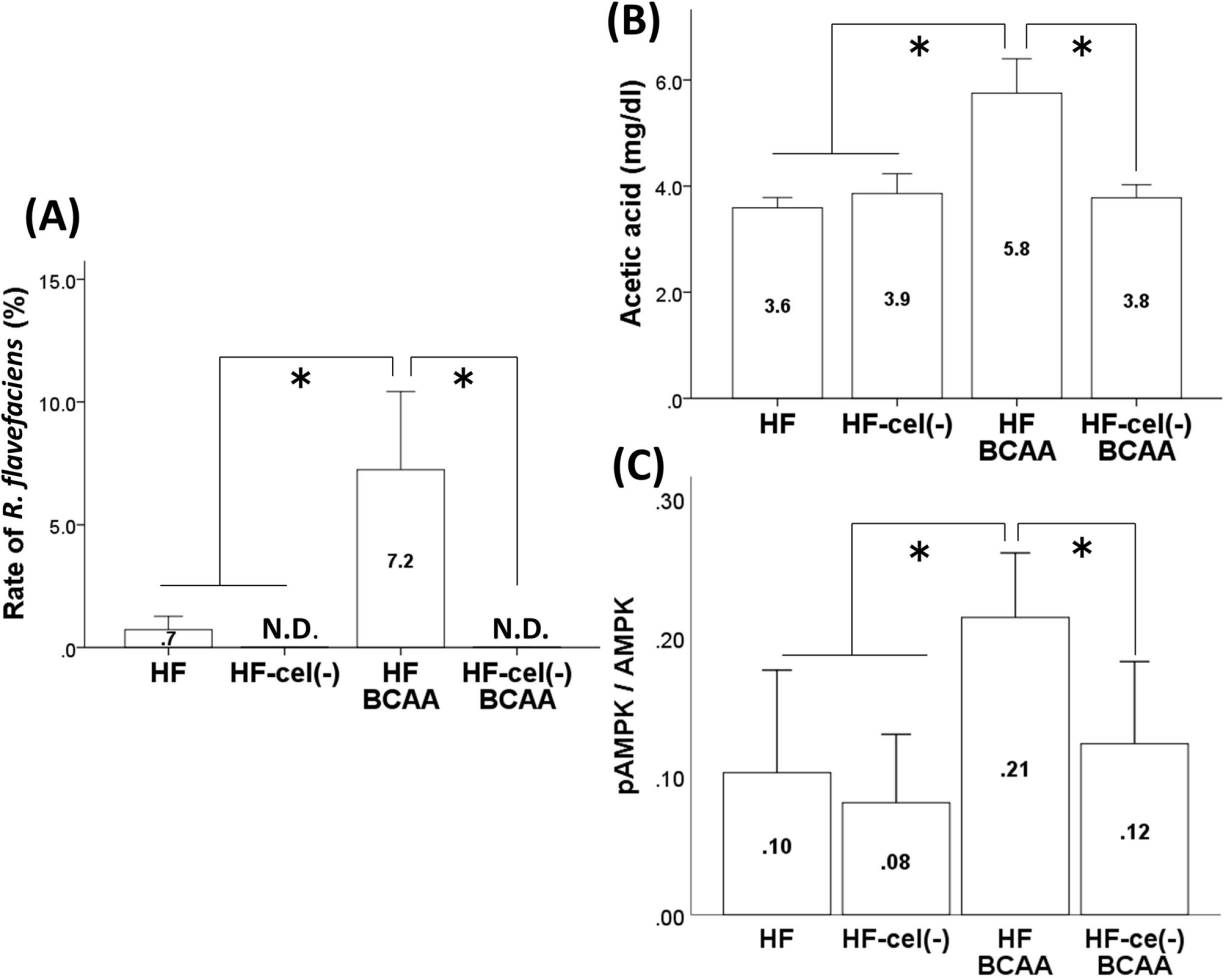
Effects of cellulose on the BCAA-induced alteration of the *R. flavefaciens* rate (**A**), portal acetic acid concentrations (**B**) and hepatic AMPK activity (**C**). Values are expressed as the means ± S.D. *p < 0.05 between the groups connected by a line. HF, fed a high-fat diet; HF-cel(–), fed a HF without cellulose; HF-BCAA, fed a HF with added BCAAs; and HF-cel(–)-BCAA, fed a cellulose-free HF with added BCAAs.

Furthermore, we investigated the role of *R. flavefaciens* in the BCAA-induced alteration in hepatic fat accumulation. In Design 2, we examined whether the proliferation of *R. flavefaciens* induced by BCAA treatment was altered in cellulose-deficient feeding because *R. flavefaciens* proliferates mainly using cellulose as a substrate. At the genus level in Design 2, BCAA treatment increased the proportion of the genus *Ruminococcus* and this finding was disappeared in conditions of cellulose deficiency (Supplementary fig. S4). At the species level, we observed that the BCAA-induced increase in the *R. flavefaciens* proportion was suppressed by cellulose deficiency, indicating that cellulose is necessary for *R. flavefaciens* to proliferate (Fig. 3A, Supplementary fig. S4).

Moreover, the concentration of acetic acid in portal vein blood for each respective group [HF group, 3.59 ± 0.10 mg/dl; HF-cel(−) group, 3.86 ± 0.19 mg/dl; HF-BCAA group, 5.75 ± 0.32 mg/dl; and HF-cel(−)-BCAA group, 3.78 ± 0.12 mg/dl] indicated that cellulose deficiency inhibited BCAA-induced elevation of portal acetic acid levels and that the proliferation of *R. flavefaciens* is related to the increase in portal acetic acid levels caused by BCAA supplementation (Fig. 3B).

### Effect of BCAA supplementation on the hepatic pAMPK/AMPK ratio in cellulose-deficient HF feeding conditions

In Design 2, the BCAA-induced increase in pAMPK/AMPK ratio in the liver was disappeared due to cellulose deficiency, suggesting that cellulose is needed to promote the hepatic activity of AMPK by BCAA supplementation in HF feeding conditions (Fig. 3C).

### Effect of BCAA supplementation on hepatic fat accumulation and liver fibrosis in cellulose-deficient HF feeding conditions

**Figure. 4:**
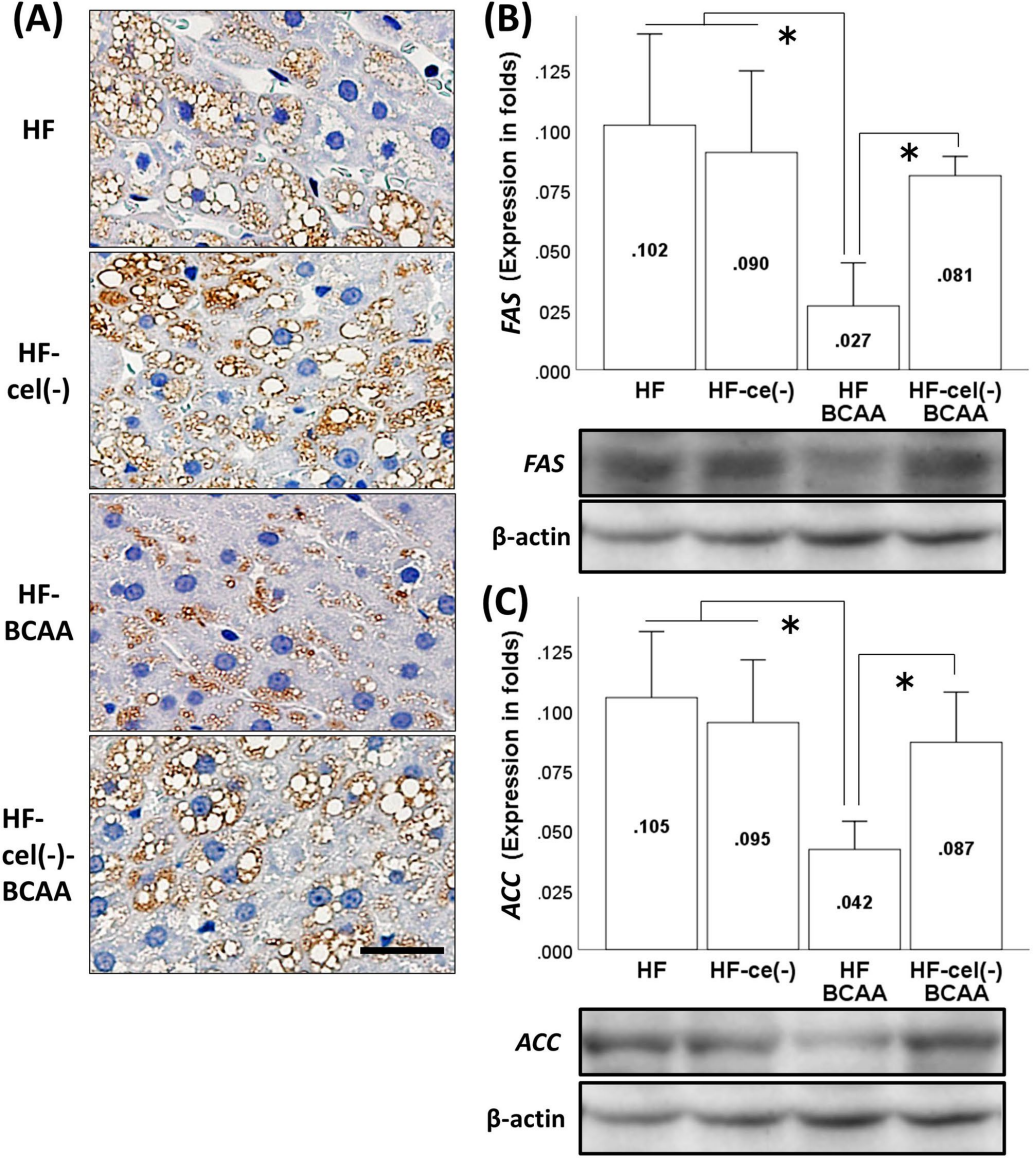
Effects of cellulose on the BCAA-induced reduction of hepatic fat accumulation (**A**) and hepatic lipogenesis-related protein expression of FAS (**B**) and ACC (**C**). Representative adipophilin immunostaining of the liver in each group (**A**). Values are expressed as the means ± S. D (**B, C**). *p < 0.05 between the groups connected by a line. Scale bar = 20 μm. A representative band of each group was trimmed. Full-length gels are presented in Supplementary figure. S10. HF, fed a high-fat diet; HF-cel(−), fed a HF without cellulose; HF-BCAA, fed a HF with added BCAAs; and HF-cel(−)-BCAA, fed a cellulose-free HF with added BCAAs.

In Design 2, the morphological findings (Fig. 4A) and results of hepatic TG content analysis (Supplementary fig. S5) show that cellulose deficiency suppressed BCAA-induced decrease in fat accumulation in the liver, although cellulose deficiency itself did not alter fat accumulation in the liver. BCAA treatment also reduced the steatosis score, but this improvement disappeared in cellulose deficient conditions, indicating that cellulose is needed to improve hepatic steatosis by BCAA administration (Supplementary fig. S5). Similar to Design 1, we did not observe liver fibrosis in each group (Supplementary fig. S5).

### Effect of BCAA administration on the expression of lipogenesis-related genes and proteins in cellulose-deficient HF feeding conditions

We observed that cellulose deficiency attenuated the BCAA-induced decrease in the transcription of *FAS* and *ACC* in the HF feeding group (Supplementary fig. S6). We also evaluated the protein expression of FAS and ACC in the liver. Furthermore, we found that cellulose deficiency repressed the BCAA-induced reduction in the protein levels of FAS (Fig. 4B, Supplementary fig. S10) and ACC (Fig. 4C, Supplementary fig. S10), which is consistent with the transcriptional changes observed for these mRNAs, suggesting that cellulose is necessary for BCAA treatment to reduce hepatic fat accumulation.

## Discussion

In this study, we examined whether BCAA supplementation alters the development of hepatic fat accumulation through alterations of intestinal microbial flora. BCAA supplementation induced the proliferation of *R. flavefaciens* and the increase of portal levels of acetic acids synthesized from *R. flavefaciens* improves HF-induced hepatic fat accumulation by downregulating the expression of FAS and ACC in the liver.

We tested supplementation with BCAA at a concentration of 3%, because other studies indicated that 3% BCAA supplementation reduced the amount of hepatic triglyceride accumulation and the expression of inflammatory cytokines in the liver as well as drug-induced liver fibrosis ^17,21,22^. Using terminal restriction fragment polymorphism (T-RFLP) analysis and next-generation sequencing, we found that *R. flavefaciens,* which produces acetic acid primarily from cellulose, was more abundant in HF feeding conditions with BCAA supplementation, indicating that BCAAs might be necessary to increase the proportion of *R. flavefaciens* ^23–25^. Several laboratories have reported a relationship between BCAAs and *R. flavefaciens*. In herbivores, BCAAs are synthesized from branched-chain volatile fatty acids (BCVFAs) by microorganisms in the stomach, and these BVCFAs are necessary for the increase in cellulolytic bacteria such as *R. flavefaciens* ^26,27^. Moreover, they promote the decomposition of dietary fibre and the growth of bacteria ^18^. In studies in which BCAAs have been removed from cell culture medium to induce intragastric fermentation in vitro, bacterial proliferation was inhibited ^19^. Moreover, when the same amounts of BCAAs and BCVFAs were added, BCAAs were found to promote the decomposition of dietary fibre in the stomach by bacteria more effectively ^20^. In this study, we have revealed that oral administration of BCAAs promotes the proliferation of *R. flavefaciens,* which is a cellulolytic bacterium. On the other hand, we observed that the genus *Coprococcus* was significantly decreased by HF feeding conditions. The genus *Coprococcus* produces butyric acid, mainly in the intestinal tract of its host, using carbohydrates as a substrate ^28^. It has been reported that the genus *Coprococcus* is less abundant in patients with NAFLD than it is in healthy controls, which is consistent with our data ^29^. Moreover, a relatively low abundance of the genus *Coprococcus* was also observed in patients with other inflammatory conditions, suggesting that a low abundance of the genus *Coprococcus* can promote chronic inflammation that may contribute to NAFLD pathogenesis ^30^.

Here, the question arises as to why BCAA administration does not lead to proliferation of *R. flavefaciens* in ST-fed rats. We found that the proportion of the genus *Coprococcus* (12.9%) was increased more than that of *R. flavefaciens* (3.6%) in the ST, although the proportion of *R. flavefaciens* (8.9%) was more abundant than that of the genus *Coprococcus* (6.7%) in the HF conditions. One possible explanation is that the ST contains 60% carbohydrates, which the genus *Coprococcus* uses as a substrate, whereas the HF only contains 35% carbohydrate. Thus, we speculate that the genus *Coprococcus* might dominate the intestinal flora in the ST, and the BCAA-induced proliferation of *R. flavefaciens* might be masked by the increase in the genus *Coprococcus*. In addition, the present study showed no difference in body weight between the ST and HF groups. There is a report that eight week of intake of a 60% HF does not induce obesity in young rats, but the total calorie intake is increased in HF feeding, which is consistent with our results ^31^. While there was a trend for the HF-fed groups to have a higher body weight (Supplementary table S1), it was not significant probably due to the relatively short duration of HF feeding.

Several studies have demonstrated that SCFAs contribute greatly to energy homeostasis and lipid metabolism in multiple tissues ^32,33^. Furthermore, SCFAs are well known for their anti-inflammatory effects, such as suppressing inflammatory reactions in the intestines, although it is uncertain how the anti-inflammatory effects of SCFAs are connected to systemic metabolic inflammation in obesity ^34^. One interesting note is that acetic acid-administered obese rats had less hepatic lipid accumulation than water-administered obese rats ^10^. When acetic acid is orally administered, it is immediately taken up from the intestine, excreted into the bloodstream, and absorbed by the tissues. Considering our results in which BCAA supplementation increased portal acetic acid levels in HF-fed rats and reduced HF-induced fat accumulation in the liver as well as improved steatosis, one part of the NAFLD activity score, BCAA administration might be useful for suppressing the development of NAFLD by acetic acid produced through intestinal bacteria such as *R. flavefaciens*.

There was no significant alteration in the plasma BCAA levels in Design 1. Amino acids, including BCAAs, exist as free amino acids inside the body and are called the "amino-acid pool". The consistency of the plasma concentration of amino acids is maintained by the "amino-acid pool", and excess amino acids are oxidatively decomposed ^35^. Therefore, the plasma concentration of BCAAs was not significantly altered by BCAA supplementation in Design 1.

As already mentioned, cellulose is essential for maintaining the proportion of *R. flavefaciens* and for producing acetic acid from *R. flavefaciens*. To further understand the impact of the proliferation of *R. flavefaciens* by BCAA administration on the elevation of portal acetic acid levels and fat accumulation in the liver, we examined whether the downregulation of *R. flavefaciens* proliferation by cellulose deficiency influences BCAA-induced proliferation of *R. flavefaciens*, elevation of portal acetic acid levels and reduction of hepatic fat accumulation. We observed that BCAA-induced increases in *R. flavefaciens* in the intestine and portal acetic acid levels were not observed in the cellulose-free diet. Similarly, the BCAA-induced reduction of fat accumulation and steatosis in the liver disappeared with cellulose deficiency, indicating that *R. flavefaciens* might play a role in inhibiting the development of NAFLD by BCAA administration. This is the first report showing that acetic acid produced by *R. flavefaciens*, which requires cellulose to proliferate in the intestinal tract, might be associated with the mechanism of action by which BCAAs alleviate fat accumulation in the liver.

The acetic acid transported to the liver is activated to acetyl-CoA with the concomitant formation of AMP. The increase in AMP concentration leads to an increase in the AMP/ATP ratio following the phosphorylation of AMPK, which is its active form in the liver. AMPK acts as the key metabolic “master switch” and regulates a number of enzymes involved in lipid homeostasis. It has been reported that the activation of AMPK leads to the inactivation of carbohydrate response element (ChRE)-binding protein (ChREBP). ChREBP upregulates two key liver lipogenic enzymes: *ACC* and *FAS*^36^. Therefore, the inhibition of lipogenesis by acetic acid might be assumed to occur in the liver through the activation of AMPK ^8^. We investigated the effect of acetic acid transported to the liver through the portal vein on hepatic fat accumulation and its mechanism, mainly focusing on lipogenesis. In this study, we clarified that the oral administration of BCAAs, which elevates portal acetic acid levels by increasing the rate of *R. flavefaciens,* activates hepatic AMPK and downregulates the transcription of *FAS* and *ACC* which are lipogenesis genes and it activates the expression of these proteins in the liver. There are some similar changes in the liver in previous studies, and oral administration of SCFAs decreased *FAS* expression as well as fat accumulation in pigs ^37^. In addition, oral administration of acetic acid to obese rats reduced the expression of lipogenesis genes such as *FAS* and *ACC*, and the intake of acetic acid contributed to lowering the accumulation of abdominal fat and protecting against the accumulation of lipids in the liver ^5,38^. On the other hand, this study showed that the portal acetic acid level was increased by 1.5 ~ 2.0 mg/dl with BCAA supplementation compared to that of the HF-fed group, which might seem to be a slight alteration. However, a previous study reported that 150 µM acetic acid increased AMPK activity due to an increase in the AMP content of primary hepatocytes. Acetic acid at concentration of 150 µM is expected to have a potency equivalent to the 0.9 mg/dl plasma acetic level, indicating that the BCAA-induced increase in the portal acetic acid concentration observed in this study is sufficient to reduce fat accumulation in the liver.

In the present study, we demonstrate that BCAA-induced reduction of hepatic fat accumulation occurs at an early stage of NAFLD based on chronic overnutrition for eight weeks. This animal model reproduces the early stage of liver disease, as shown by steatosis with low inflammation and no fibrosis. From a methodological point of view, this is of paramount importance, since the pathogenic mechanisms responsible for the transition from steatosis to steatohepatitis and fibrosis in early stage NAFLD could be a potential therapeutic target. Furthermore, we used a 45% fat diet to produce early- stage NAFLD because other studies have demonstrated that BCAA supplementation ameliorates obesity induced by a 45% fat diet but not by a 60% fat diet ^17,39^. These findings indicate that the significant effects of BCAAs appear to be sensitive to fat levels in the diet.

Our study has some limitations. First, the low number of samples may influence the results. Second, differences in types of high-fat diets such as diets containing 60% or 45% fat may influence the gut microbiota. Third, regarding faecal sample collection, there was variability among many studies, which may affect the gut microbiota. Currently, the gut microbiota has been widely recognized as a remarkable factor that leads to the development of obesity, although disparities exist. Differences in data analysis techniques and reference databases could result in discrepancies, which were excluded from this study. However, different ages of animals and different commercial dealers are also potential confounding factors that may affect a component of microbiota, which is a limitation of this study ^40^. In addition, different 16S rRNA gene regions used in individual datasets may cause differences in taxonomic categories. In the future, more studies under consistent conditions should be performed.

**Figure. 5:**
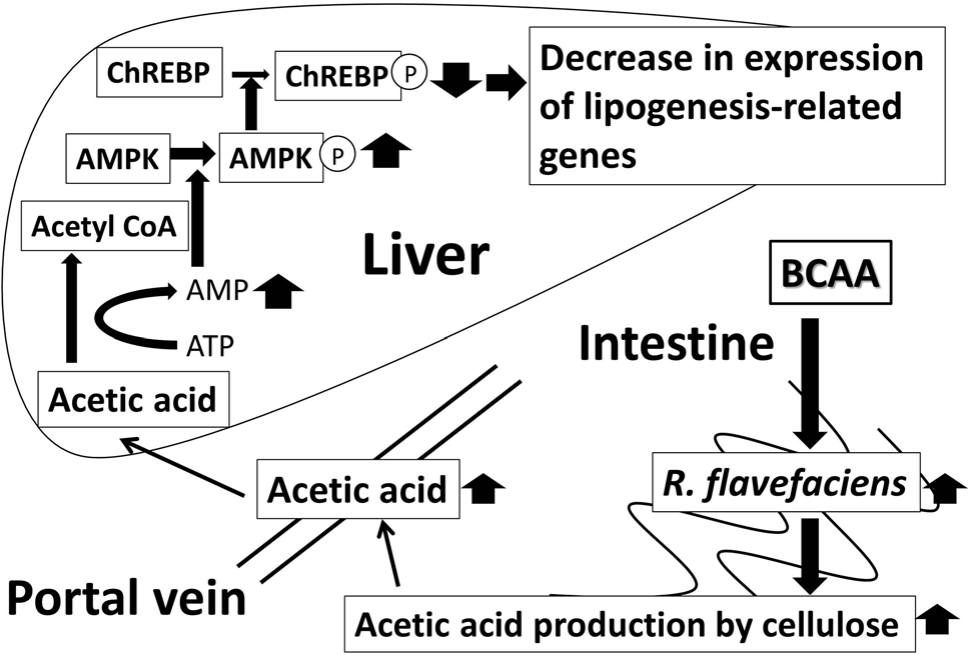
Mechanism of action by which BCAAs improve NAFLD. The administration of BCAAs promotes *R. flavefaciens* proliferation and subsequent synthesis of acetic acid which suppresses the expression of lipogenesis-related genes in the liver.

In conclusion, we found that the administration of BCAAs promotes *R. flavefaciens* proliferation and subsequently increases the amount of acetic acid produced from *R. flavefaciens*. This acetic acid is thought to be excreted into the portal venous bloodstream and, when absorbed in the liver and metabolized, is thought to suppress the expression of lipogenesis-related genes, thereby reducing fat accumulation in the liver (Fig. 5). These findings indicate that BCAAs may be useful in preventing the development of NAFLD, although further studies are needed to assess this hypothesis.

## Material and methods

### Animals

Male 8-week-old Sprague–Dawley rats (270–330 g; Charles River Laboratories, Inc., Kanagawa, Japan) were housed in individual cages in a room with daily illumination from 07:00 to 19:00 (12-h/12-h light/dark cycle) and were maintained at 21 ± 1 °C with 55 ± 5% humidity. Animals were provided ad libitum access to chow and tap water. All experiments were performed in accordance with the guidelines established by the National Institutes of Health, USA, regarding the care and use of animals for experimental procedures. Additionally, the ethics committee of the Division of Laboratory Animal Science, Research Promotion Project of Oita University, specifically approved this study.

### Diets

The basal diet consisted of six types: a standard diet (ST; 10% fat, 70% carbohydrate, 20% protein; Research Diets, Inc., New Brunswick, NJ, USA), a ST plus BCAA diet (ST-BCAA; 10% fat, 67% carbohydrate, 23% protein), a high-fat diet (HF; 45% fat, 35% carbohydrate, 20% protein; Research Diets, Inc.), a HF plus BCAA diet (HF-BCAA; HF; 45% fat, 33% carbohydrate, 22% protein), a HF without cellulose diet [HF-cel(-); 45% fat, 35% carbohydrate, 20% protein] and a cellulose-free HF plus BCAA diet [HF-cel(-)-BCAA; 45% fat, 33% carbohydrate, 22% protein]. The ingredients and nutrient compositions of each diet are shown in Supplementary fig. S7. The absolute amounts of leucine, isoleucine and valine per 100 g diet supplemented with BCAAs were 2.7 ~ 3.4 g, 1.5 ~ 1.8 g, and 2.0 ~ 2.5 g, respectively.

### Experimental protocol

*Design 1* Twenty-four rats were assigned to one of four groups. In group 1 (ST group, n = 6), rats were fed the standard diet for 8 weeks. In group 2 (ST-BCAA group, n = 6), rats were fed a ST supplemented with BCAAs (accounting for 3% of the total kcals) for 8 weeks. In group 3 (HF group, n = 6), rats were fed a HF for 8 weeks. In group 4 (HF-BCAA group, n = 6), rats were fed a HF supplemented with BCAAs (accounting for 3% of the total kcals) for 8 weeks. The daily dietary intake, body weight and the increase in body weight during the experiment were measured.

*Design 2* Twenty-four rats were assigned to one of four groups. In group 1 (HF group, n = 6), rats were fed a HF for 8 weeks. In group 2 (HF-cel(-) group, n = 6), rats were fed a HF without cellulose for 8 weeks. In group 3 (HF-BCAA group, n = 6), rats were fed a HF supplemented with BCAAs (accounting for 3% of the total kcals) for 8 weeks. In group 4 (HF-cel(-)-BCAA group, n = 6), rats were fed a cellulose-free HF supplemented with BCAAs (accounting for 3% of the total kcals) for 8 weeks. The daily dietary intake, body weight and increase in body weight during the experiment were measured.

The protocols of Designs 1 and 2 are shown in the Supplementary fig. S8.

### 16S metagenomics

The faecal samples were lysed by incubating the samples in lysis buffer, and these were homogenized by bead beating with zirconium beads (0.1 mm and 0.3 mm) using a TissueLyser (Qiagen, Tokyo, Japan) for 15 min at a speed of 25 m/s. Then, DNA was extracted from each 20-mg ileum stool sample using a QIAamp DNA Stool Mini Kit (Qiagen), in accordance with the method described by the manufacturer. Forward and reverse primers were prepared as described by Caporaso et al. ^41^, and the 16S V4 region of the DNA was PCR amplified using TaKaRa Ex Taq (Takara Bio, Inc., Siga, Japan). Thereafter, we purified the amplified DNA using a DNA purification kit (Qiagen) according to the method described by the manufacturer. We measured the DNA concentration of each sample by RT-PCR using a Light Cycler (Roche Diagnostics K.K., Tokyo, Japan) with a SYBR Green PCR kit (Qiagen), and we created a calibration curve using DNA standards (KAPA Biosystems, Wilmington, MA, USA) and equalizing the concentration to 1000 ng/ml. The equalized samples were subjected to electrophoresis using a 1% agarose gel with a 20-bp DNA ladder (Takara Bio, Inc.) as a control, and we ensured that there were no unnecessary bands and that there was a band of approximately 250 bp. We prepared a mixture containing 2-μl of each sample to generate a mixed library using a MiSeq reagent kit v2 (Illumina, Inc., San Diego, CA, USA) for MiSeq sequencing. We prepared a mixture of 200 μl of library solution (5 μl of mixed library, 5 μl of 0.2 N NaOH and 990 μl of HT1 buffer), 150 μl of PhiX solution (2 μl of 10 nM PhiX control v3 (Illumina, Inc.), 3 μl of EB buffer (Qiagen), 0.5 μl of 0.2 N NaOH, and 990 μl of HT1 buffer) and 250 μl of HT1 buffer as a final sample to be analysed. Read 1, read 2 and index primers were prepared as described by Caporaso et al. ^41^. The library was subjected to primary analysis using MiSeq (Illumina, Inc.), while secondary analysis was performed using the sequencing hub application “16S metagenomics v1.0”, which is provided on “Base Space”, a cloud service provided by Illumina, Inc.

### Measurement of portal acetic acid levels and hepatic triglyceride (TG) contents

After anaesthetization with sodium pentobarbital (50 mg/kg, i.p.), blood was obtained from the portal vein of each rat and it was centrifuged at 4000×*g* for 10 min at 4 °C. Serum was immediately frozen and stored at − 80 °C until analysis. Finally, the rats were exsanguinated following transcardial perfusion with 100 ml of saline containing 200 U of heparin. Livers were removed, immediately frozen and stored at − 80 °C until analysis. The concentration of short-chain fatty acids was measured in portal vein blood with gas chromatography tandem mass spectrometry using GCMS-QP2020 (Shimadzu Corporation, Kyoto, Japan). The area from the baseline of the obtained waveform was used as an indicator of the concentration of acetic acid. The concentration was calculated based on a calibration curve created using step diluted acetic acid. Liver tissue (100 mg) was homogenized for 10 min in 1 ml of a solution containing 150 mM NaCl, 0.1% Triton X-100, and 10 mM Tris using a Polytron homogenizer (NS-310E; Micro Tech Nichion, Chiba, Japan). Liver TG contents was measured using an automatic analyser (SRL, Tokyo, Japan).

### Histological analyses

Liver tissue samples were fixed with 4% paraformaldehyde and then were embedded in paraffin. Sections of 5-µm thickness were cut with a vibratome, stained with haematoxylin and eosin (HE) and examined under a microscope (Olympus, Tokyo, Japan). For assessing liver fibrosis, reticulin staining using silver impregnation was performed to detect reticulin fibres. We observed the tissue stained with HE and generated a score for steatosis and fibrosis of the NAFLD activity score (NAS). The score was defined from medium-power evaluation (100× magnification) of parenchymal involvement by steatosis as follows: 0 (< 5%), 1 (5–33%), 2 (34–66%), and 3 (> 66%). Each sample was scored based on 10 views, and the mean score was calculated. The fibrosis score was also defined as follows: 0 (none), 1 (pericellular fibrosis), 2 (sinusoid and pericellular), 3 (bridging fibrosis), and 4 (cirrhosis).

We performed tissue immunostaining with adipophilin to stain the membrane of the lipid droplet. We used an adipophilin rabbit polyclonal antibody (LifeSpan BioSciences Inc., Seattle, WA, USA) as the primary antibody and completed the immunohistochemical staining with a biotin-labelled secondary antibody.

### RT-PCR

Total RNA was isolated from liver tissue using an RNeasy mini kit (Qiagen) in accordance with the method described by the manufacturer and reverse-transcribed to obtain cDNA using the PrimeScript RT Reagent Kit (Takara Bio, Inc.).

2 μg cDNA was used for relative quantitative PCR analysis with specific primers such as *ACC*, *FAS*, and *β-actin*. The sequences of the primers were as follows: *ACC* (NM022193, sense: CCTTCTTCTACTGGCGACTGAG, antisense: TAAGCCTTCACTGTGCCTTCC), *FAS* (NM017332, sense: GAGGACTTGGGTGCCGATTAC, antisense: GCTGTGGATGATGTTGATGATAGAC), and *β-actin* (NM031144, sense: GGCACCACACTTTCTACAAT, antisense: AGGTCTCAAACATGATCTGG). RT-PCR was performed for 40 cycles using the following cycling conditions and TB Green Premix Ex Taq II (Takara Bio, Inc.): 30 s of denaturation at 95 °C, 5 s of annealing at 95 °C and 30 s of extension at 60 °C.

### Western blotting

Frozen liver tissue preparations were homogenized with sodium dodecyl sulfate (SDS) sample buffer, clarified by centrifugation, and boiled. The total protein concentration in each tissue sample was quantified by the Bradford method, and 8 µg of total protein per sample was loaded onto 8% SDS–polyacrylamide gels for electrophoresis (PAGE). The separated proteins were transferred onto polyvinylidene difluoride (PVDF) membranes (Bio-Rad Laboratories). The membranes were incubated with a primary antibody solution consisting of 5 g/L polyclonal antiserum with specificity for FAS, ACC and β-actin. These primary antibodies were purchased from Santa Cruz Biotechnology (Texas, USA). Immunoreactive bands were detected by enhanced chemiluminescence (Amersham Life Science, Buckinghamshire, UK) and were quantified using National Institutes of Health imaging software. We measured the FAS, ACC and β-actin of each specimen and determined each protein level as a ratio to that of β-actin between each group.

### AMPK and pAMPK in ELISA

We measured total AMPK and pAMPK levels using the following kits in accordance with the method described by the manufacturer: rat phosphorylated AMP-activated protein kinase ELISA kit (MBS7230575; MyBioSource, Inc., San Diego, CA, USA) for measuring pAMPK levels, and rat total AMP-activated protein kinase (AMPK) ELISA kit (MBS1602983; MyBioSource, Inc.) for measuring AMPK levels.

### Biochemical data

Serum alanine aminotransferase (ALT) and total cholesterol (TC) were measured using an automatic analyser (RINTEC, Japan). Serum triglyceride (TG) and glucose levels were measured with each assay kit (Wako, Tokyo, Japan). Serum amino acid levels such as BCAAs were measured with liquid chromatography using a JLC-500 (JEOL Ltd., Tokyo, Japan) fully automated amino acid analyser.

### Statistical analysis

Data were analysed by one-way analysis of variance and Tukey’s or Fischer’s post hoc test using SPSS, and p < 0.05 was assumed to indicate statistical significance.

## Supplementary information

Supplementary Information

## Data Availability

All data generated or analysed during this study are included in this article and its supplementary information.
