# Supplementary material for: Supplementation of branched-chain amino acids decreases fat accumulation in the liver through intestinal microbiota-mediated production of acetic acid
Source: Sci Rep. 2020 Oct 30;10:18768. doi: 10.1038/s41598-020-75542-3 (PMC7603487; doi:10.1038/s41598-020-75542-3)
Supplement: Supplementary file 1 — Supplementary Information [file 41598_2020_75542_MOESM1_ESM.pdf]

Supplementary information

Supplementation of branched-chain amino acids decreases fat accumulation in the liver through intestinal microbiota-mediated production of acetic acid

Masao Iwao, Koro Gotoh, Mie Arakawa, Mizuki Endo, Koichi Honda, Masataka Seike, Kazunari Murakami and Hirotaka Shibata.

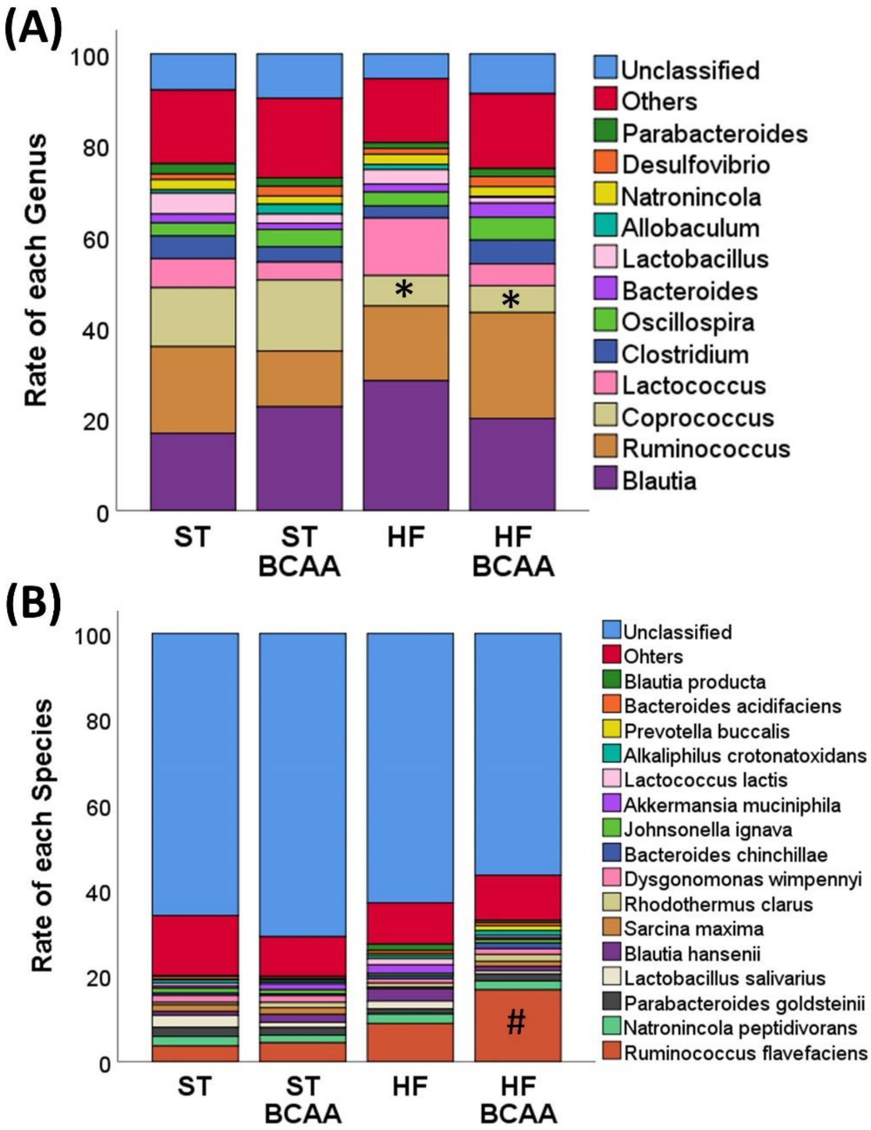

Supplementary figure. S1. Proportion of each genus (A) and species (B) of intestinal flora in Design 1.

*, $p < 0.05$ vs ST and ST-BCAA; # $p < 0.05$ vs ST, ST-BCAA and HF

ST, fed a standard diet; ST-BCAA, fed ST with added BCAAs; HF, fed a high-fat diet; and HF-BCAA, fed a HF with added BCAAs.

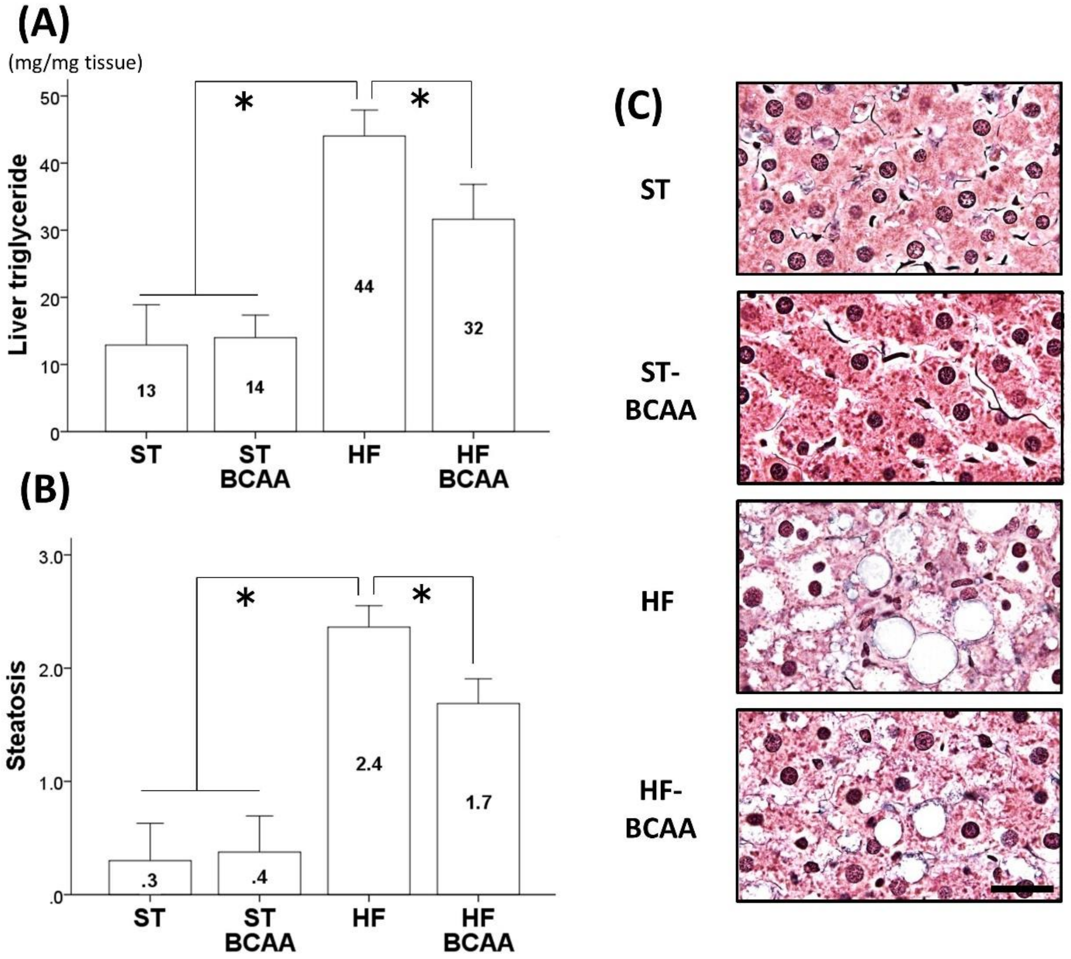

Supplementary figure. S2. Effects of BCAAs on the HF-induced fat accumulation and fibrosis in the liver.

Contents of hepatic triglyceride (A) and steatosis, which is one of measures included in the NAFLD activity score (B). Representative reticulin staining using silver impregnation of liver in each group (C). Values are expressed as the means ± S.D. *, $p < 0.05$ between the groups connected by a line. Scale bar = 20 μm.

ST, fed a standard diet; ST-BCAA, fed a ST with added BCAAs; HF, fed a high-fat diet; and HF-BCAA, fed a HF with added BCAAs.

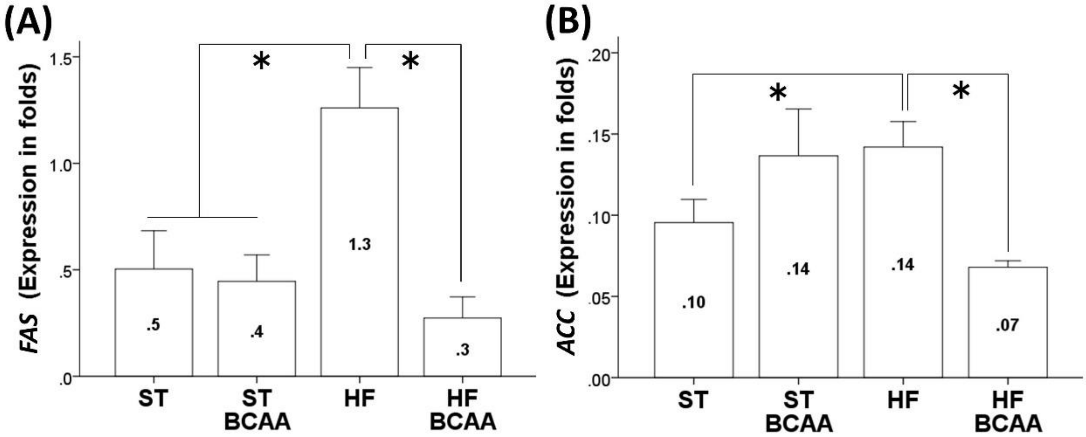

Supplementary figure. S3. Effects of BCAAs on HF-induced hepatic lipogenesis-related gene transcription of *FAS* (A) and *ACC* (B).

*, $p < 0.05$ between the groups connected by a line

ST, fed the standard diet; ST-BCAA, fed ST with added BCAAs; HF, fed a high-fat diet; and HF-BCAA, fed HF with added BCAAs.

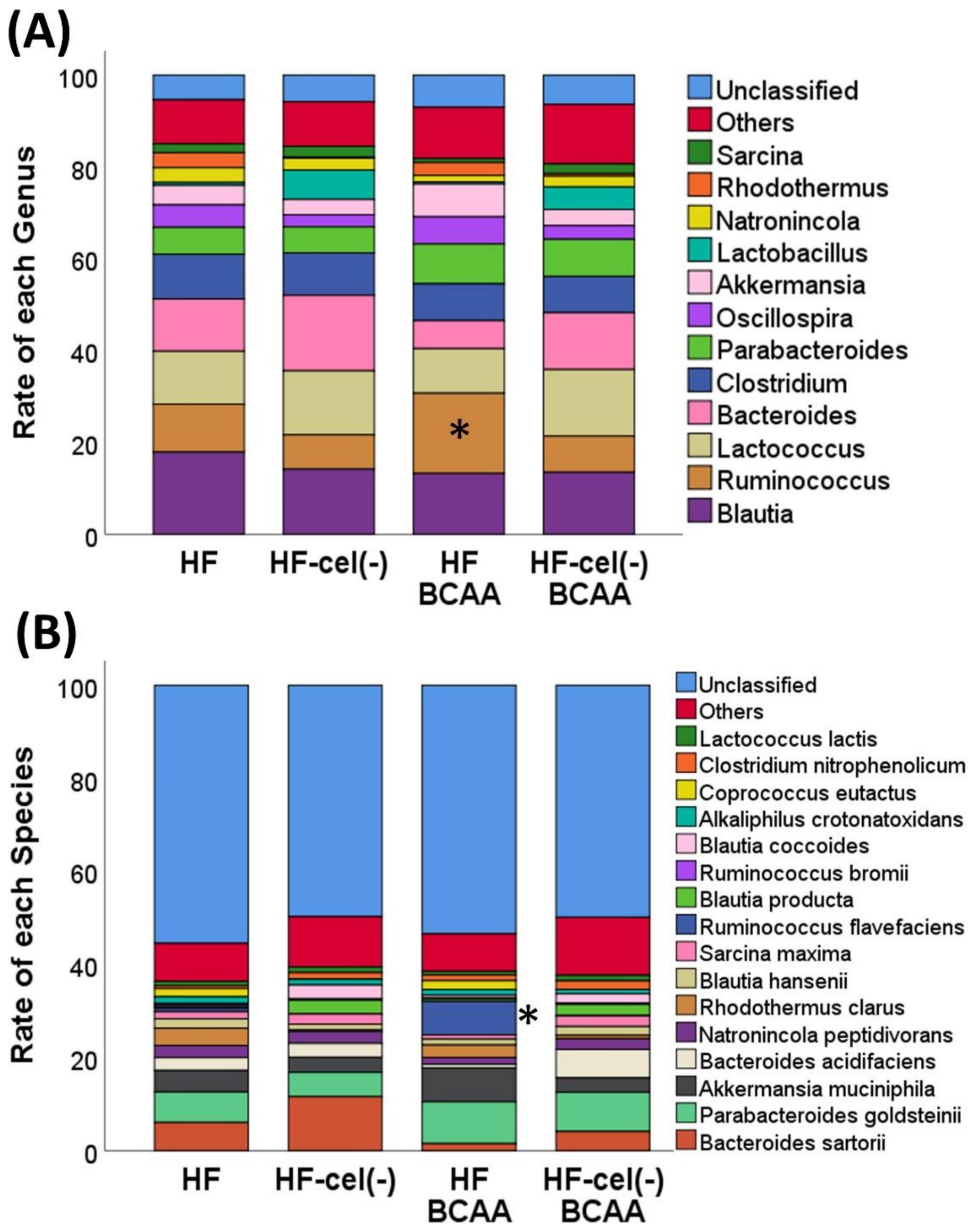

Supplementary figure. S4. Rate of each genus (A) and species (B) of intestinal flora in Design 2.

*, $p < 0.05$ vs other groups

HF, fed a high-fat diet; HF-cel(-), fed a HF without cellulose; HF-BCAA, fed a HF with added BCAAs; and HF-cel(-)-BCAA, fed a cellulose-free HF with added BCAAs.

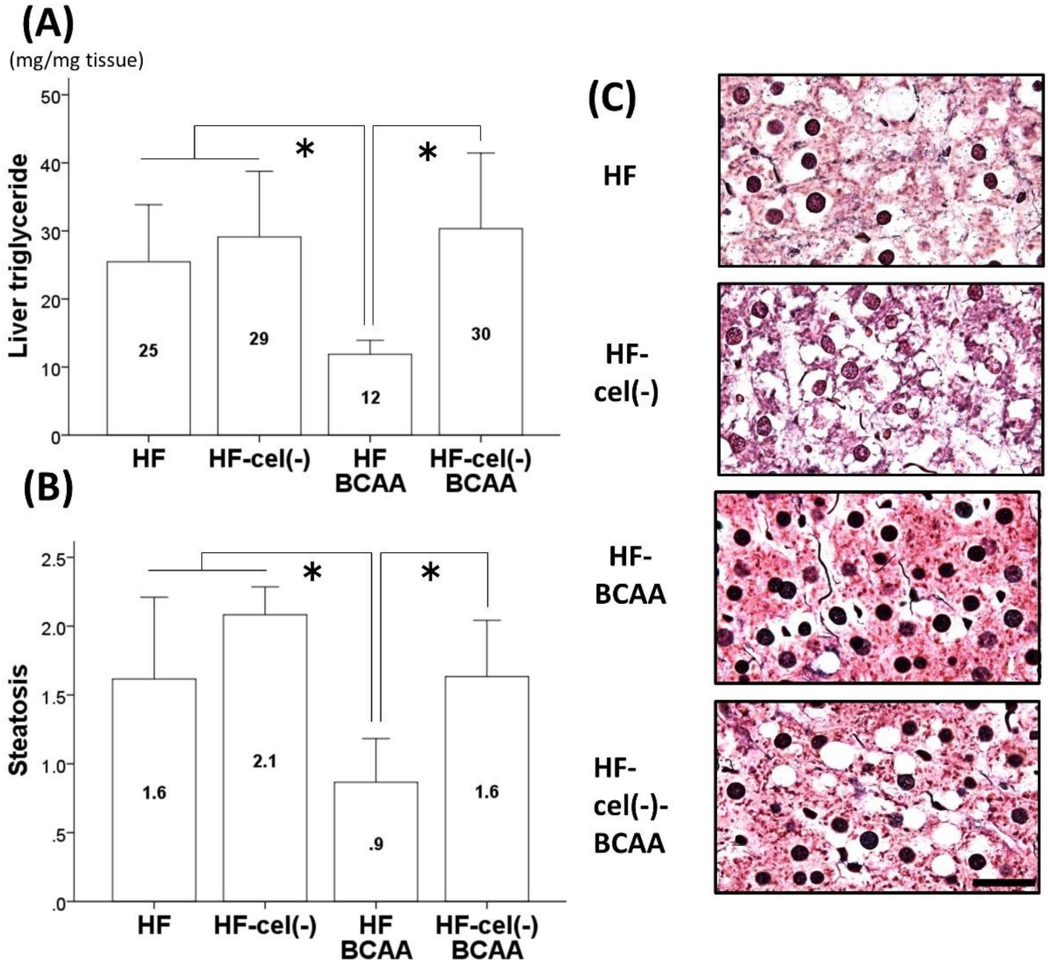

Supplementary figure. S5. Effects of cellulose on the BCAA-induced reduction of hepatic fat accumulation and fibrosis

Contents of hepatic triglyceride (A) and steatosis, which is one of measures included in the NAFLD activity score (B). Representative reticulin staining using silver impregnation of liver in each group (C). Values are expressed as the means ± S.D. *, $p < 0.05$ between the groups connected by a line. Scale bar = 20 μm.

HF, fed a high-fat diet; HF-cel(-), fed a HF without cellulose; HF-BCAA, fed a HF with added BCAAs; and HF-cel(-)-BCAA, fed a cellulose-free HF with added BCAAs.

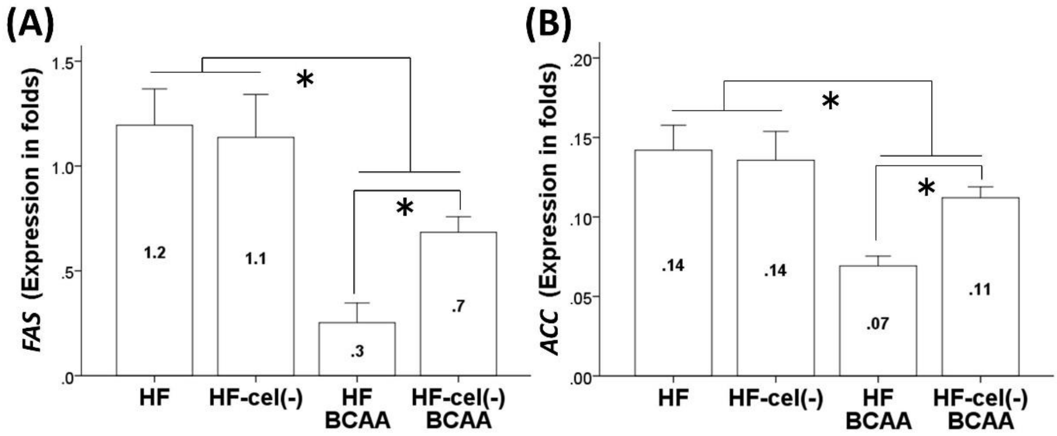

Supplementary figure. S6. Effects of cellulose on the BCAA-induced improvement in hepatic lipogenesis-related gene transcription of *FAS* (A) and *ACC* (B).

*, $p < 0.05$ between the groups connected by a line

HF, fed a high-fat diet; HF-cel(-), fed a HF without cellulose; HF-BCAA, fed a HF with added BCAAs; and HF-cel(-)-BCAA, fed a cellulose-free HF with added BCAAs.

Diet	ST		ST-BCAA		HF		HF-BCAA		HF-cel(-)		HF-cel(-)-BCAA	
	gm%	kcal%	gm%	kcal%	gm%	kcal%	gm%	kcal%	gm%	kcal%	gm%	kcal%
Protein	19	20	22	23	24	20	27	22	25	20	28	22
Carbohydrate	67	70	65	67	41	35	38	33	44	35	41	33
Fat	4	10	4	10	24	45	24	45	25	45	25	45
Total		100		100		100		100		100		100
kcal/gm	3.8		3.8		4.7		4.7		4.7		4.7	
**Ingredient**	**gm**	**kcal**	**gm**	**kcal**	**gm**	**kcal**	**gm**	**kcal**	**gm**	**kcal**	**gm**	**kcal**
Casein without BCAAs	159	646	159	646	159	646	159	646	159	646	159	646
Leucine, L	17	58	29.2	107	17	58	29.2	107	17	58	29.2	107
Isoleuscine, L	10	40	16.1	64	10	40	16.1	64	10	40	16.1	64
Valine, L	14	56	21.4	86	14	56	21.4	86	14	56	21.4	86
L-Cystine	3	12	3	12	3	12	3	12	3	12	3	12
Corn Starch	452.2	1809	426.5	1706	72.8	291	47.1	188	72.8	291	47.1	188
Maltodextrin 10	75	300	75	300	100	400	100	400	100	400	100	400
Sucrose	172.8	691	172.8	691	172.8	691	172.8	691	172.8	691	172.8	691
Cellulose	50	0	50	0	50	0	50	0	0	0	0	0
Soybean Oil	25	225	25	225	25	225	25	225	25	225	25	225
Lard	20	180	20	180	177.5	1598	177.5	1598	177.5	1598	177.5	1598
Meneral Mix	10	0	10	0	10	0	10	0	10	0	10	0
DiCalcium Phosphate	13	0	13	0	13	0	13	0	13	0	13	0
Calcium Carbonate	5.5	0	5.5	0	5.5	0	5.5	0	5.5	0	5.5	0
Potassium Citrate	16.5	0	16.5	0	16.5	0	16.5	0	16.5	0	16.5	0
Vitamin Mix	10	40	10	40	10	40	10	40	10	40	10	40
Choline Bitartrate	2	0	2	0	2	0	2	0	2	0	2	0
Yellow Dye	0.04	0	0.025	0	0	0	0	0	0.025	0	0.04	0
Red Dye	0.01	0	0	0	0.05	0	0	0	0.025	0	0	0
Blue Dye	0	0	0.025	0	0	0	0.05	0	0	0	0.01	0
**Total**	**1055.5**	**4057**	**1055.05**	**4057**	**858.15**	**4057**	**858.15**	**4057**	**858.15**	**4057**	**858.15**	**4057**

Supplementary figure. S7. Ingredients and nutrient compositions of each diet.

ST, fed a standard diet; ST-BCAA, fed a ST with added BCAAs; HF, fed a high-fat diet; HF-BCAA, fed a HF with added BCAAs; HF-cel(-), fed a HF without cellulose; and HF-cel(-)-BCAA, fed a cellulose-free HF with added BCAAs.

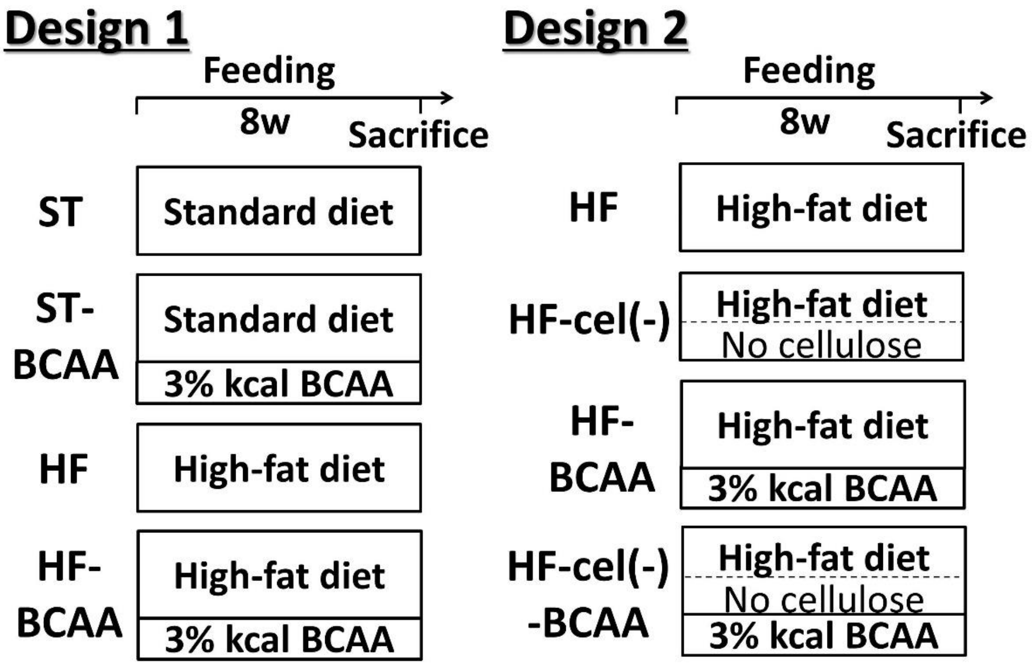

Supplementary figure. S8. Protocol of experimental Designs 1 and 2.

ST, fed a standard diet; ST-BCAA, fed a ST with added BCAAs; HF, fed a high-fat diet; HF-BCAA, fed a HF with added BCAAs; HF-cel(-), fed a HF without cellulose; and HF-cel(-)-BCAA, fed a cellulose-free HF with added BCAAs.

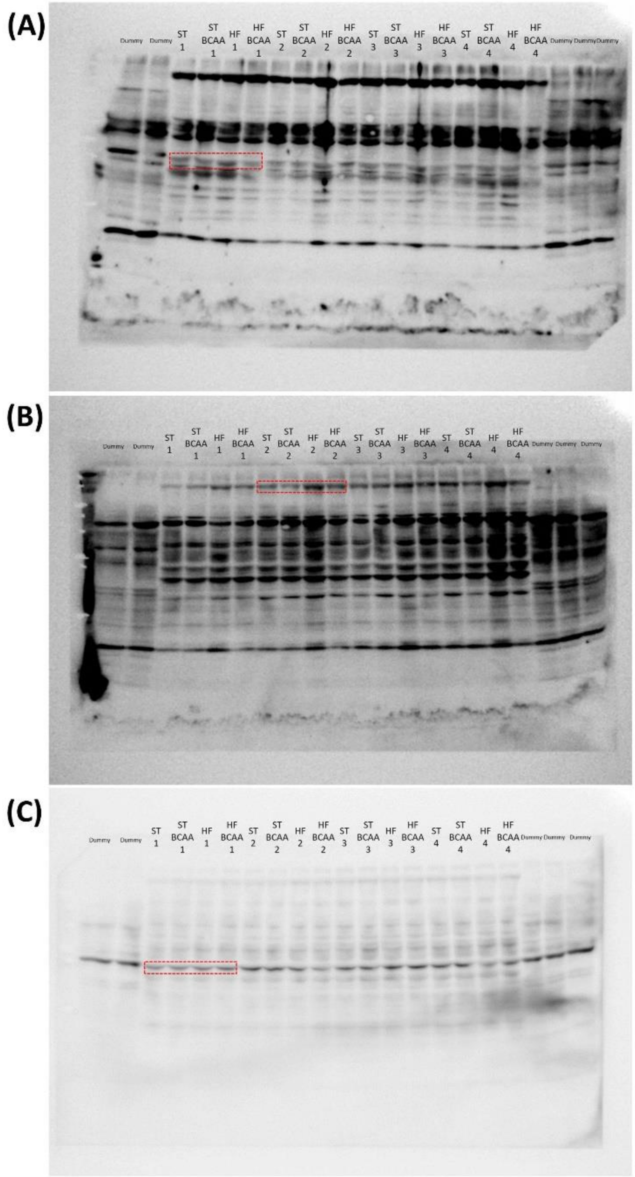

Supplementary figure. S9. Full-length western blot gels of FAS (A), ACC (B) and β-actin (C) in Design 1.

The samples derive from the same experiment and those gels were processed in parallel. The trimmed area is indicated by the red box line.

ST, fed the standard diet; ST-BCAA, fed ST with added BCAAs; HF, fed a high-fat diet; and HF-BCAA, fed HF with added BCAAs.

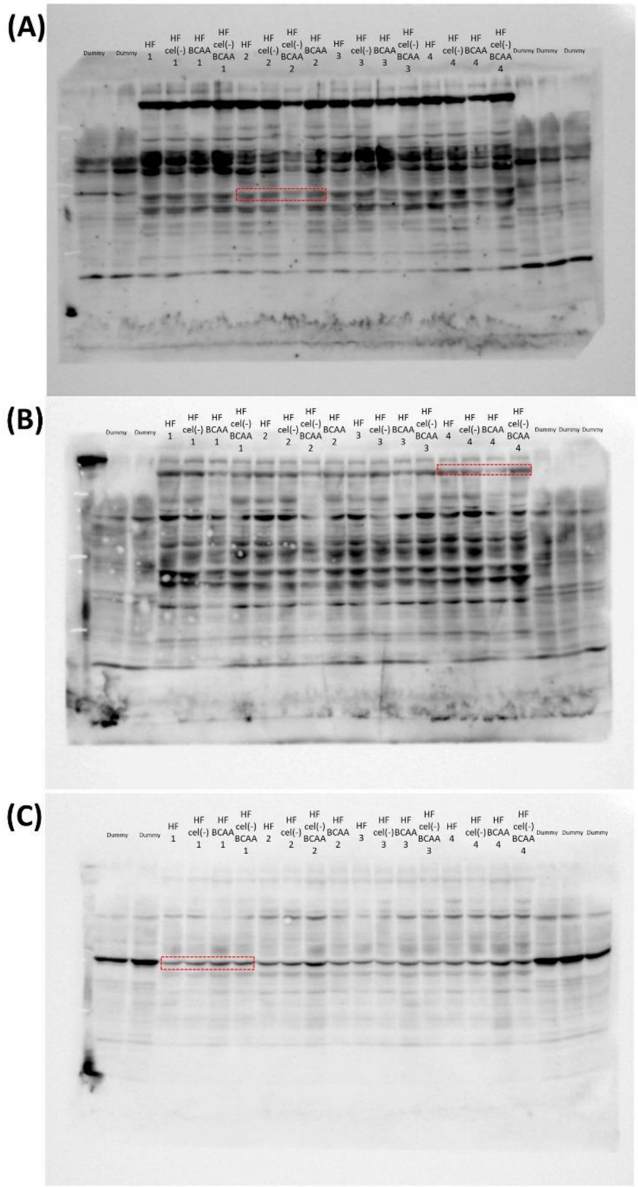

Supplementary figure. S10. Full-length western blot gels of FAS (A), ACC (B) and β-actin (C) in Design 2.

The samples derive from the same experiment and those gels were processed in parallel. The trimmed area is indicated by the red box line.

HF, fed a high-fat diet; HF-cel(-), fed a HF without cellulose; HF-BCAA, fed a HF with added BCAAs; and HF-cel(-)-BCAA, fed a cellulose-free HF with added BCAAs.

Design 1	ST	ST-BCAA	HF	HF-BCAA
**Daily intake (kcal)**	95.3 ± 2.4	98.2 ± 3.1	106.3± 3.5	113.8 ± 4.6 *
**Body weight (g)**	598.2 ± 13.3	589.3 ± 16.3	642.0 ± 18.3	641.2 ± 17.3
**Serum BCAA (μg/ml)**	69.9 ± 2.7	73.1 ± 4.3	62.6 ± 1.5	71.8 ± 2.1
**ALT (U/L)**	33.1 ±5.1	49.5 ± 15.0	33.6 ± 4.9	31.4 ± 4.9
**Total-chol (mg/dL)**	86.3 ± 5.3	85.3 ± 8.6	70.8 ± 3.1	80.6 ± 5.4
**Triglyceride (mg/dL)**	139.7 ± 8.6	124.9 ± 11.3	114.1 ± 14.8	126.8 ± 30.7
**Glucose (mg/dL)**	130.1 ± 10.8	128.7 ± 14.1	143.7 ± 5.6	142.9 ± 5.8

Supplementary table. S1. Daily intake, body weight and biochemical data after 8 weeks of feeding in each group of Designs 1.

*, $p < 0.05$ vs. ST, ST-BCAA. Total-chol: total cholesterol.

Design 2	HF	HF-cel (-)	HF-BCAA	HF-cel (-)-BCAA
**Daily intake (kcal)**	113.5 ± 4.0	115.1 ± 3.8	109.1 ± 2.8	117.6 ± 4.7
**Body weight (g)**	696.2 ± 17.4	688.2 ± 16.1	665.4 ± 7.9	662.8 ± 10.5
**ALT (U/L)**	31.4 ±1.8	33.1 ± 2.2	27.7 ± 2.4	27.5 ± 1.8
**Total-chol (mg/dL)**	101.5 ± 6.8	102.3 ± 8.2	93.9 ± 9.4	107.1 ± 5.1
**Triglyceride (mg/dL)**	129.8 ± 8.0	130.2 ± 13.4	127.0 ± 9.9	121.9 ± 15.9
**Glucose (mg/dL)**	153.9 ± 10.9	144.5 ± 7.1	153.3 ± 7.3	158.3 ± 9.0

Supplementary table. S2. Daily intake, body weight and biochemical data after 8 weeks of feeding in each group of Designs 2.

Total-chol: total cholesterol.
